# Tuberculosis of the Air-Passages above the Larynx

**Published:** 1910-12-03

**Authors:** 


### Laryngology and Rhinology.

### TUBERCULOSIS OF THE AIR-PASSAGES ABOVE THE LARYNX.

The larynx is so frequently and so seriously involved in tuberculosis that it is apt to overshadow the other parts of the upper respiratory tract, the tuberculous affections of which are nevertheless of sufficient importance to merit consideration.

In addition to true tuberculosis, lupus also occurs in the upper respiratory tract, and the two affections are at times very liable to be confused. It is probable that lupus is primarily the result of infection of the nasal mucosa by tubercle bacilli conveyed thither in the air or on the finger, and that the unfavourable environment so reduces the virulence of the organism that it is able only to produce the modified lesions known as lupus; from the nasal cavities the disease spreads forwards on to the skin and backwards to the palate, fauces, and larynx. On the other hand, tuberculosis of these regions is in the large majority of cases the result of infection spreading upwards from the lungs. The larynx is attacked before the pharynx, while the nares are only very rarely affected at all; only some 2 per cent. of patients who have died of phthisis show the lesions of tuberculosis within the nose.

Tuberculosis of the nose is, however, very commonly described; it is frequently primary, is chronic in its course, is usually found in young women and most often originates at the lower and anterior part of the septum, within reach of the finger-nail. The lesions of lupus and tuberculosis in the nose are, in fact, absolutely indistinguishable, and it is therefore quite unnecessary to retain the double classification; as the clinical features agree with those of lupus rather than of tuberculosis it is better to classify the affection under the former heading.

In the larynx and pharynx both diseases exist and are readily distinguishable. The characteristics of laryngeal lupus are in complete contrast to those of tuberculosis; it affects the margin of the epiglottis first and spreads thence to the lumen of the organ; it is chronic and painless in its course, tends to heal with the formation of much dense cicatricial tissue, and shows the characteristic nodular infiltration. On the pharynx, too, lupus is distinct, affecting first the upper surface and margin of the soft palate and spreading thence to the faucial pillars and pharyngeal wall, and showing throughout a striking absence of pain.

True tuberculosis of the pharynx occurs almost always in the late stage of pulmonary phthisis, when the patient's resistance is much broken down and when the larynx is already secondarily diseased. The affection is rapid or even fulminating in its course; the most vulnerable parts are the pillars of the fauces, but the disease spreads rapidly and involves the palate, pharyngeal wall, and even the insides of the cheeks in a diffuse infiltration, which rapidly breaks down with the formation of extensive superficial ulceration. Miliary tubercles are often seen around the ulcers, and these in turn soon break down and coalesce, and so the ulceration spreads; the palate may be pale or reddened, and is frequently very œdematous and so thickened as to become immobile. The characteristic of the disease is intense dysphagia, so that the unfortunate patient prefers starvation to the severe pain caused by any attempt to swallow.

Except in some cases of disease limited to the posterior wall, all attempts to stay the progress of the disease are completely unsuccessful, and most of those attacked die within a few weeks. Unfortunately, too, treatment directed to the relief of pain is very unsatisfactory, much less successful than in the larynx. Orthoform and anæsthesin, in the form of insufflation, give some relief, but these powders do not stay in contact with the parts as well as in the larynx. Resort must generally be made to cocaine, which in these cases may be used *ad libitum* and in increasing concentration. If the pain is at all localised, curetting of the ulcer affords most relief, and sometimes the application of solid nitrate of silver makes the surface less sensitive. Hypodermic injections of morphia usually become the only remedy of any avail.

Besides this acute form of pharyngeal tuberculosis, the lymphoid tissue of the upper air-passages is often affected in consumptive patients; the lesions are seldom apparent clinically, but can only be discovered by microscopical examination. This latent tuberculosis affects the faucial and pharyngeal tonsils, and also is said to attack the lingual tonsil and the lymphoid nodules on the posterior pharyngeal wall. Statistics on this point vary considerably, but it would appear that tuberculous lesions may be demonstrated in the tonsils in well over half the cases of patients who have died of consumption. In addition to these secondary cases, tonsils and adenoids contain tubercle bacilli or microscopic tubercles in a small number of persons who show no signs of tuberculosis elsewhere, and this opens up the very large subject of the portal of entry of the tubercle bacillus into the body; there is no doubt that the cervical lymphatic glands may become tuberculous through tonsils which show no apparent clinical signs of tuberculosis, but it is doubtful whether the lungs can be infected directly through the cervical lymphatic chain and the apical pleura. Several isolated cases of clinically obvious primary tuberculosis of the faucial tonsils have also been recorded.

